# Sleep Duration and Incidence of Cardiovascular Events in a Japanese Population: The Jichi Medical School Cohort Study

**DOI:** 10.2188/jea.JE20090053

**Published:** 2010-03-05

**Authors:** Yoko Amagai, Shizukiyo Ishikawa, Tadao Gotoh, Kazunori Kayaba, Yosikazu Nakamura, Eiji Kajii

**Affiliations:** 1 Division of Community and Family Medicine, Center for Community Medicine, Jichi Medical University, Shimotsuke, Tochigi, Japan; 2 Wara National Health Insurance Clinic, Gujo, Gifu, Japan; 3 School of Health and Social Services, Saitama Prefectural University, Koshigaya, Saitama, Japan; 4 Department of Public Health, Jichi Medical University, Shimotsuke, Tochigi, Japan

**Keywords:** cohort studies, sleep, cardiovascular disease, cerebrovascular disease

## Abstract

**Background:**

Although sleep is one of the most important health-related factors, the relationship between sleep duration and the incidence of cardiovascular events has not been fully described.

**Methods:**

The present study comprised the 11 367 study subjects (4413 men and 6954 women) of the Jichi Medical School Cohort Study, a population-based prospective study. Baseline data were obtained by questionnaire and health examinations between April 1992 and July 1995 in 12 rural areas in Japan, and the main outcome measures were the incidence of cardiovascular diseases (stroke and myocardial infarction [MI]). Cox proportional hazards models were used to analyze the association between sleep duration and the incidence of cardiovascular events.

**Results:**

A total of 481carciovascular events (255 men and 226 women) were observed during an average follow-up period of 10.7 years. After adjusting for age, systolic blood pressure, serum total cholesterol, body mass index, smoking habits, and alcohol drinking habits, the hazard ratios (95% confidence intervals) for the incidence of cardiovascular diseases for individuals sleeping less than 6 hours and 9 hours or longer were 2.14 (1.11–4.13) and 1.33 (0.93–1.92) in men, and 1.46 (0.70–3.04) and 1.28 (0.88–1.87) in women, respectively, relative to those who reported sleeping 7 to 7.9 hours per day.

**Conclusions:**

Our data indicate that men who sleep less than 6 hours a day have a higher risk of cardiovascular events than those sleeping 7 to 7.9 hours.

## INTRODUCTION

Sleep is one of the most important factors contributing to health; however, sleep loss, long-term sleep deprivation, and alterations in sleep duration are common in modern society. The factors responsible for this change include increased environmental light, increased shift and night work, and the advent of television, radio, and the Internet.^1^

Various studies have examined the relationship between sleep duration and mortality. Most reports have found a U-shaped relationship between sleep duration and all-cause mortality in both men and women.^2^^–^^6^ We also reported that men with short sleep duration and women with long sleep duration had an elevated risk of death.^7^ However, few studies have examined the relationship between sleep duration and the incidence of cardiovascular diseases,^8^^–^^11^ especially in Asian people, who, as compared to Western populations, often have a lower body mass index (BMI) and different lifestyle and dietary risk factors for cardiovascular diseases. The purpose of the present study was to examine whether sleep duration is associated with the incidence of cardiovascular diseases, ie, stroke and myocardial infarction (MI), in a Japanese population.

## METHODS

### Study population

We used the data set of the Jichi Medical School Cohort Study, a population-based prospective study that was started in 1992 to investigate the risk factors for cardiovascular diseases.^12^ The baseline data were obtained between April 1992 and July 1995 in 12 rural areas in Japan. Mass screening examinations for cardiovascular diseases have been conducted since 1983 in accordance with the Health and Medical Service Law for the Aged of 1982, and we used this system to collect the data. The subjects for mass screening examinations were residents aged 40 to 69 years in 8 areas, those aged 30 years or older in 1 area, and subjects of different ages in 3 other areas. In each community, the local government office sent personal invitations to all subjects by mail. In total, 12 490 people (4911 men and 7579 women) were eligible. The overall response rate among the 12 communities was 65%. Written informed consent to participate in the study was obtained individually from all the respondents to the mass screening.

Among the 12 490 participants, we excluded 95 (0.8%) who did not sign the agreement to participate in the study and 7 (0.06%) who had no follow-up data, after which 12 388 subjects (4869 men and 7519 women) remained. For the present study, we identified 4509 men and 7028 women with data on sleep duration, and excluded those who reported a past history of MI (40 men and 25 women) or stroke (62 men and 50 women). Ultimately, 11 367 subjects (4413 men and 6954 women) aged 18 to 90 years remained.

### Measurement of baseline variables

The health checkup was carried out in each community. BMI was calculated as weight (kg)/height (m)^2^. Systolic blood pressure (SBP) and diastolic blood pressure (DBP) were measured with a fully automated sphygmomanometer (BP203RV-II, Nippon Colin, Komaki, Japan). Serum total cholesterol and high density lipoprotein (HDL) cholesterol were also measured using standard methods, as reported previously.^12^

Information about the subjects’ medical history and sociodemographic characteristics was obtained by trained interviewers using a standardized questionnaire. Smoking status was classified as current smoking, former smoking, or never smoking, and alcohol drinking status was classified as current drinking, former drinking, or never drinking. Participants were asked what time they usually fell asleep at night and awoke in the morning. This enabled us to calculate sleep duration.

### Follow-up

Repeat examinations, which are also a part of the national mass screening program, were used to follow up most subjects every year. Subjects were asked whether they had a history of stroke or coronary heart diseases after enrolling in the study. Those with such histories were asked the name of the hospital they had visited. Subjects who did not undergo the screening examination were contacted by mail or phone. Medical records were checked if the subjects were hospitalized for any reason. Public health nurses also visited the subjects’ home to obtain further information.

If an incident case of stroke or MI was suspected, duplicate computed tomography films or magnetic resonance imaging films (for stroke), or electrocardiograms (for MI) were requested. Data on subjects who moved out of the study area were obtained annually from the municipal government.

### Diagnostic criteria

Diagnosis was determined independently by a diagnosis committee comprising a radiologist, a neurologist, and 2 cardiologists. Criteria for stroke were a focal and nonconvulsive neurological deficit of sudden onset persisting for 24 hours or longer; stroke subtype was determined by using the criteria of the National Institute of Neurological Disorder and Stroke. A diagnosis of MI was determined by using the criteria of the World Health Organization Multinational Monitoring of Trends and Determinants in Cardiovascular Disease (MONICA) Project, which was a multinational collaborative project to monitor coronary events from the mid-1980s to the mid-1990s.

### Statistical analysis

Statistical analysis was performed using SPSS for Windows, version 11.5 (SPSS Inc., Japan). Descriptive parameters are shown as the mean, standard deviation, or proportion (%). We compared characteristics between groups by 1-way analysis of variance or the chi-square test. Cox proportional hazards models were used to calculate hazard ratios for the incidence of cardiovascular diseases, after adjusting for age, SBP, serum total cholesterol level, BMI, smoking habits, and alcohol drinking habits, which were considered to be potential confounding factors.

This study was approved by the Institutional Review Board of Jichi Medical University.

## RESULTS

The mean follow-up period ± standard deviation (SD) was 10.7 ± 2.4 years. The mean age ± SD was 55.1 ± 11.9 years for men and 55.3 ± 11.2 for women at baseline. A total of 411 strokes and 80 MIs were confirmed during the follow-up period. There were 207 strokes and 55 MIs among men, and 204 strokes and 25 MIs among women.

At baseline, 2.9% of men and 3.7% of women slept less than 6 hours a day, 10.8% and 16.1% slept 6 to 6.9 hours, 28.5% and 36.6% slept 7 to 7.9 hours, 37.5% and 31.8% slept 8 to 8.9 hours, and 20.3% and 11.8% slept 9 hours or longer. The mean ± SD of sleep duration was 7.8 ± 1.2 hours in men and 7.4 ± 1.1 hours in women. The baseline characteristics in relation to each category of sleep duration are shown in Table 1. Mean age, SBP, DBP, and serum total cholesterol level at baseline were positively associated with sleep duration in both sexes. Mean HDL cholesterol level was inversely associated with sleep duration in women.

**Table 1.** Baseline characteristics of subjects, by sleep duration

Sex		Sleep duration (hours/day)										*P*-value

		–5.9		6.0–6.9		7.0–7.9		8.0–8.9		9.0–		
Men	*n* (%)	126	(2.9)	478	(10.8)	1258	(28.5)	1653	(37.5)	898	(20.3)	
	Age (years)	53.3	(14.8)	51.4	(11.8)	52.6	(11.8)	55.3	(11.5)	60.4	(10.6)	<0.001^a^
	Systolic blood pressure (mm Hg)	130.4	(19.1)	129.4	(20.8)	129.6	(19.8)	131.2	(20.4)	135.3	(21.3)	<0.001^a^
	Diastolic blood pressure (mm Hg)	78.2	(11.8)	78.1	(13.0)	78.3	(12.3)	79.2	(12.3)	81.0	(12.0)	<0.001^a^
	Total cholesterol (mg/dL)	184.7	(33.5)	184.5	(34.2)	186.3	(34.3)	186.0	(33.8)	181.7	(34.9)	0.023^a^
	High-density lipoprotein cholesterol (mg/dL)	48.8	(12.8)	47.5	(12.5)	48.9	(14.1)	48.9	(13.3)	49.7	(13.3)	0.103^a^
	Body mass index (kg/m^2^)	22.9	(2.8)	23.0	(2.9)	23.1	(2.8)	22.9	(2.9)	22.7	(2.9)	0.055^a^
	Current smokers (%)	56.0		54.5		50.2		52.3		45.2		0.002^b^
	Current alcohol drinkers (%)	72.8		72.6		75.0		78.3		72.5		0.007^b^

Women	*n* (%)	256	(3.7)	1118	(16.1)	2548	(36.6)	2213	(31.8)	819	(11.8)	
	Age (years)	52.6	(10.5)	52.4	(10.8)	53.3	(11.0)	57.1	(10.9)	61.4	(10.2)	<0.001^a^
	Systolic blood pressure (mm Hg)	123.4	(17.9)	125.3	(20.6)	126.8	(21.1)	129.3	(20.8)	133.8	(21.7)	<0.001^a^
	Diastolic blood pressure (mm Hg)	74.3	(10.3)	75.2	(12.2)	75.7	(12.3)	76.8	(11.9)	78.7	(11.9)	<0.001^a^
	Total cholesterol (mg/dL)	193.7	(36.6)	194.2	(35.6)	196.0	(35.2)	198.7	(33.8)	200.5	(33.9)	<0.001^a^
	High-density lipoprotein cholesterol (mg/dL)	53.0	(12.5)	53.5	(12.9)	53.0	(12.4)	52.5	(12.3)	51.1	(12.7)	0.001^a^
	Body mass index (kg/m^2^)	23.2	(3.2)	23.3	(3.1)	23.0	(3.1)	23.1	(3.2)	23.5	(3.5)	0.002^a^
	Current smokers (%)	8.8		6.7		5.5		4.9		4.5		0.021^b^
	Current alcohol drinkers (%)	26.2		28.6		25.9		23.3		20.9		0.001^b^

Data are expressed as mean values (standard deviation) or as percentages.

^a^Analysis of variance (ANOVA).

^b^Chi-square test (degrees of freedom = 4).

Table 2 shows the crude incidence rate and hazard ratios for cardiovascular diseases by sleep duration. The crude incidence rate was higher in men who slept less than 6 hours/day or 9 hours/day or longer. After adjusting for age, men sleeping less than 6 hours/day or 9/day hours or longer had a significantly higher risk than did those sleeping 7 to 7.9 hours. After further adjustment for SBP, serum total cholesterol level, BMI, smoking habit, and alcohol drinking habit, the results were essentially unaltered, but the hazard ratio for men sleeping 9 hours or longer was no longer significant. In women, the crude incidence rate was higher among those who slept 9 hours or longer. However, there was no significant association between sleep duration and cardiovascular diseases, even among women who slept 9 hours or longer.

**Table 2.** Association between sleep duration and incidence of cardiovascular diseases: crude incidence rate and adjusted hazard ratios

Sex	Sleep duration (hours/day)	Number of cases (cases/total (%))		Crude incidence rate^a^	HR-age (95% CI)	HR-all (95% CI)
Men	–5.9	11/126	(8.7)	8.7	1.98 (1.03–3.79)	2.14 (1.11–4.13)
	6.0–6.9	20/478	(4.2)	4.0	1.11 (0.66–1.86)	1.04 (0.61–1.76)
	7.0–7.9	52/1258	(4.1)	3.9	1.00 (reference)	1.00 (reference)
	8.0–8.9	85/1653	(5.1)	4.8	1.04 (0.74–1.47)	0.98 (0.69–1.40)
	9.0–	87/898	(9.7)	9.6	1.50 (1.05–2.12)	1.33 (0.93–1.92)

Women	–5.9	9/256	(3.5)	3.3	1.47 (0.73–2.94)	1.46 (0.70–3.04)
	6.0–6.9	20/1118	(1.8)	1.6	0.71 (0.43–1.17)	0.64 (0.38–1.10)
	7.0–7.9	69/2548	(2.7)	2.5	1.00 (reference)	1.00 (reference)
	8.0–8.9	72/2213	(3.3)	3.0	0.87 (0.63–1.22)	0.85 (0.60–1.20)
	9.0–	56/819	(6.8)	6.5	1.37 (0.95–1.97)	1.28 (0.88–1.87)

HR-age: hazard ratios adjusted for age.

HR-all: hazard ratios adjusted for age, systolic blood pressure, total cholesterol, body mass index, smoking habits, and alcohol drinking habits.

^a^per 1000 person-years.

We also analyzed separately the association between sleep duration and the incidences of stroke and MI (Tables 3 and 4). For men, the crude incidence rate and the hazard ratio for stroke were essentially the same as those detailed above, although the age-adjusted risk for men who slept 9 hours or longer was significantly higher than that of men who slept 7 to 7.9 hours. For women, there was no material association between sleep duration and stroke. The risk of MI was higher in women who slept less than 6 hours.

**Table 3.** Association between sleep duration and incidence of stroke: crude incidence rate and adjusted hazard ratios

Sex	Sleep duration (hours/day)	Number of cases (cases/total (%))		Crude incidence rate^a^	HR-age (95% CI)	HR-all (95% CI)
Men	–5.9	8/126	(2.4)	6.3	1.84 (0.86–3.94)	2.00 (0.93–4.31)
	6.0–6.9	17/478	(0.8)	3.4	1.24 (0.70–2.18)	1.13 (0.63–2.03)
	7.0–7.9	40/1258	(1.2)	3.0	1.00 (reference)	1.00 (reference)
	8.0–8.9	70/1653	(1.0)	3.9	1.11 (0.75–1.64)	1.03 (0.69–1.53)
	9.0–	72/898	(1.9)	7.9	1.60 (1.08–2.37)	1.39 (0.92–2.10)

Women	–5.9	6/256	(1.2)	2.2	1.06 (0.46–2.46)	0.97 (0.39–2.41)
	6.0–6.9	19/1118	(0.2)	1.6	0.75 (0.45–1.25)	0.68 (0.39–1.18)
	7.0–7.9	62/2548	(0.4)	2.2	1.00 (reference)	1.00 (reference)
	8.0–8.9	66/2213	(0.3)	2.7	0.89 (0.63–1.26)	0.86 (0.60–1.23)
	9.0–	51/819	(0.6)	5.9	1.39 (0.95–2.04)	1.29 (0.86–1.91)

HR-age: hazard ratios adjusted for age.

HR-all: hazard ratios adjusted for age, systolic blood pressure, total cholesterol, body mass index, smoking habits, and alcohol drinking habits.

^a^per 1000 person-years.

**Table 4.** Association between sleep duration and incidence of myocardial infarction: crude incidence rate and adjusted hazard ratios

Sex	Sleep duration (hours/day)	Number of cases (cases/total (%))		Crude incidence rate^a^	HR-age (95% CI)	HR-all (95% CI)
Men	–5.9	3/126	(2.4)	2.3	1.73 (0.50–6.06)	1.78 (0.50–6.28)
	6.0–6.9	4/478	(0.8)	0.8	0.76 (0.25–2.30)	0.77 (0.25–2.33)
	7.0–7.9	15/1258	(1.2)	1.1	1.00 (reference)	1.00 (reference)
	8.0–8.9	16/1653	(1.0)	0.9	0.69 (0.34–1.39)	0.69 (0.34–1.41)
	9.0–	17/898	(1.9)	1.8	1.01 (0.49–2.06)	0.99 (0.47–2.06)

Women	–5.9	3/256	(1.2)	1.1	3.78 (1.02–13.97)	4.93 (1.31–18.61)
	6.0–6.9	2/1118	(0.2)	0.2	0.55 (0.12–2.53)	0.59 (0.13–2.73)
	7.0–7.9	9/2548	(0.4)	0.3	1.00 (reference)	1.00 (reference)
	8.0–8.9	6/2213	(0.3)	0.2	0.53 (0.19–1.50)	0.59 (0.21–1.66)
	9.0–	5/819	(0.6)	0.6	0.83 (0.27–2.56)	0.84 (0.27–2.62)

HR-age: hazard ratios adjusted for age.

HR-all: hazard ratios adjusted for age, systolic blood pressure, total cholesterol, body mass index, smoking habits, and alcohol drinking habits.

^a^per 1000 person-years.

## DISCUSSION

We investigated the association between reported sleep duration and the incidence of cardiovascular diseases in a Japanese population from the Jichi Medical School Cohort Study, a population-based cardiovascular cohort study. We found that men sleeping less than 6 hours/day were at significantly higher risk.

Several studies have examined the relationship between sleep duration and the incidence of cardiovascular diseases. According to Qureshi et al, in the First National Health and Nutrition Examination Survey Epidemiologic Follow-up Study, stroke risk was higher in people who reported sleeping longer than 8 hours at night, as compared to those sleeping between 6 and 8 hours.^8^ In the same study, the risk for coronary heart disease was increased in individuals who slept less than 6 hours. Ayas et al reported that self-reported short and long sleep duration were independently associated with a modestly increased risk of coronary events in the Nurses’ Health Study.^9^ In the Monitoring Trends and Determinants in Cardiovascular Diseases Augsburg surveys, modest associations between short sleep duration and the incidence of MI were seen in middle-aged women, but not in men.^10^ In the Women’s Health Initiative Observational Study, Chen et al found that long sleep duration increases the risk for ischemic stroke.^11^

Several explanations for the associations between decreased sleep duration and cardiovascular diseases have been put forward. Short-term sleep deprivation caused impaired glucose tolerance, higher evening cortisol levels, activation of the sympathetic nervous system, increased blood pressure, reduced leptin levels, and increased inflammatory markers.^13^^–^^16^ Although the magnitude of the physiologic changes found in these short-term studies was modest, they provide a potential mechanism whereby long-term sleep restriction may affect long-term health. In contrast, there was no evidence suggesting that increased sleep duration affects lifespan.

We examined the incidence of stroke and MIs. In men, the crude incidence rate and the hazard ratios for stroke were essentially the same as those for cardiovascular diseases. In women, there was no material association between sleep duration and stroke, although short sleep duration was associated with a higher incidence of MI. However, the results of the present study were based on small number of incident cases, which was particularly true with MI. Thus, there were wide confidence intervals for the point estimates of the associations. Further studies including a greater number of subjects and incident cases would help determine the magnitude of the associations with greater precision.

In Japan, the Japan Collaborative Cohort Study for Evaluation of Cancer Risk reported an association between sleep duration and mortality from cardiovascular disease and other causes among Japanese men and women.^17^ As compared to 7 hours sleep, short sleep duration was associated with increased mortality from coronary heart disease in women; long sleep duration was associated with increased mortality from total stroke, ischemic stroke, and total cardiovascular disease in both men and women. As described above, our findings were based on a small number of incident cases, so significant differences were less likely to appear. However, our study did show that men sleeping less than 6 hours/day had a significantly higher risk of cardiovascular disease, whereas the Japan Collaborative Cohort Study for Evaluation of Cancer Risk showed no elevation in risk.

Our study possesses two main strengths. First, to our knowledge, it is the first such study conducted in Japan. Second, our study subjects included both men and women, who were analyzed separately.

However, there were several limitations. First, data on sleep duration and potential risk factors are self-reported by the participants. Concerns about self-reported sleep duration were raised in other studies,^18^^,^^19^ namely, are self-reported values valid in comparison with quantitative sleep assessments by actigraphy. In the current study, we asked for information about daily activities, after which we calculated sleep duration; therefore, the data were deemed to be more accurate than those that would be obtained by simply asking about sleep duration. A second weakness of the study is that we had no information on the quality of sleep in our cohort study. Several studies have reported that sleep quality was related to mortality.^4^^,^^20^^–^^22^ Indeed, only a few studies have examined the relationship between sleep duration and the incidence of cardiovascular diseases. Finally, the reasons for short sleep (insomnia, overwork, or television watching) were not ascertained in our study. These factors might have affected the risk of cardiovascular diseases.

In conclusion, our data indicate that men sleeping less than 6 hours/day had a higher risk of cardiovascular events than did those sleeping 7 to 7.9 hours.
